# Right drug, wrong dosage: insights from the PAVE-AF antithrombotic study in older patients with atrial fibrillation

**DOI:** 10.1007/s11239-020-02167-8

**Published:** 2020-06-09

**Authors:** Stylianos Tzeis, Paraskevi Savvari, Ioannis Skiadas, Sotirios Patsilinakos, Kimon Stamatelopoulos, Spyridon Kourouklis, Sotirios Kyrikos, Konstantinos Tsatiris, Damianos Menegas, George Hahalis, George Giannakoulas, the PAVE-AF study group, Dimitrios Chryssos, Georgios Diakakis, Konstantinos Gkouias, Krystallenia Kafkala, Maria Kantziou, Athanasios Kapetanopoulos, Petros Kikas, Petros Kirytopoulos, Dimitrios Korres, Efstathios Koulouris, Konstantinos Kyratlidis, Aggeliki Laina, Stylianos Lampropoulos, Georgios Lymperopoulos, Dimitrios Makrygiannis, Athanasios Maragiannis, Theodoros Michailidis, Irini Mpourni, Efthymia Nasothymiou, Christoforos Olympios, Dimitrios Papadogiannis, Eleni Paphianou, Neofytos Papoulidis, Athanasios Protogerou, Dionysia Ralli, Panagiotis Rigopoulos, Ilias Sihlimiris, Stavros Spanodimos, Christos Stathopoulos, Maria Toumpourleka, Grigorios Tsigkas, Dimitrios Tziakas, Thomas Tzimas, Nikiforos Vrettos, Tsilla Zafiriou, Aristides Zafiris, Georgios Zonios

**Affiliations:** 1 Cardiology Department, Mitera General Hospital, Hygeia Group, 6, Erithrou Stavrou, Maroussi, 15123 Athens, Greece; 2 Medical Department of Internal Medicine, Pfizer Hellas, Athens, Greece; 3 Cardiology Department, General Hospital “Agia Olga”, Athens, Greece; 4 Therapeutic Department, General Hospital “Alexandra”, Athens, Greece; 5 5th Cardiology Department, Hygeia Hospital, Athens, Greece; 6 Cardiology Department, General Hospital of Eastern Achaia-Aegio, Aegio, Greece; 7 Cardiology Department, General Hospital Karditsa, Karditsa, Greece; 8 Cardiology Department, University General Hospital Patras, Patras, Greece; 9 Cardiology Department, University General Hospital “AHEPA”, Thessaloniki, Greece

**Keywords:** Atrial fibrillation, Older, Antithrombotic, Dosage, NOAC, Anticoagulation

## Abstract

Optimal antithrombotic treatment of older patients is usually impeded by several prevailing misconceptions. The aim of our study was to assess the type, dosage and predictors of antithrombotic therapy in older patients with non-valvular atrial fibrillation (NVAF). PAVE-AF was a prospective, cross-sectional study, including NVAF patients ≥ 80 years from 30 participating centers. Demographic data, comorbidities and treatment patterns were documented in a single visit. Patients treated with non-vitamin K oral anticoagulants (NOACs) were further classified into three dosing categories (recommended, underdosing and overdosing). Among 1018 patients (85.4±4.0 years), 88.4% received anticoagulants (AC), 8% antiplatelets (AP) and 3.6% no treatment. The primary reason for AP administration was physician concern of bleeding followed by patient denial. Patients ≥90 years had two times greater probability to receive AP therapy compared to patients < 90 years. Among patients treated with AC, one third received vitamin K antagonists, while two thirds received NOACs [34.6% apixaban, 9.5% dabigatran and 22.6% rivaroxaban]. Independent predictors of AC prescription over AP or no treatment were low HAS-BLED score, hypertension, labile INR, permanent AF, absence of uncontrolled hypertension, prior stroke/systemic embolism, age and male gender. In total, 37% of NOAC recipients received inappropriate dosage, while the number of patients receiving recommended dosing differed significantly among NOAC subgroups (p < 0.001). In our study, a minority of older NVAF patients received AP or no therapy for stroke prevention. Among patients treated with anticoagulants, two thirds were on NOAC treatment, though with a considerable proportion of inappropriate dosing.

**Electronic supplementary material:**

The online version of this article (10.1007/s11239-020-02167-8) contains supplementary material, which is available to authorized users.

## Highlights

- Two thirds of patients ≥ 80 years treated with oral anticoagulants receive NOACs.
- Only 63% of NOAC recipients receive appropriate dosing.
- Patients ≥90 years have two times greater probability to receive antiplatelet therapy compared to patients < 90 years.
- Low HAS-BLED, labile INR, permanent AF, male gender, and prior stroke are independent predictors of anticoagulant prescription.
- Our results raise a red flag with respect to subtherapeutic anticoagulant dosage and point out the need for continuous education on appropriate drug therapy, irrespective of patient’s age.

## Introduction

Atrial fibrillation (AF) is the most common arrhythmia and a disease of aging. Population aging has substantially increased the total AF burden, but also the relative proportion of affected older patients. Therefore, overall improved care of AF patients is not feasible without focusing on the subgroup of older patients [1].

Stroke prevention with anticoagulants (AC) is a reasonable choice in older patients with AF. However, several prevailing misconceptions may impede optimal thromboprophylaxis. Advanced age per se has been reported as a prominent physician-related barrier to administration of AC in AF patients leading to underdosing [2, 3]. Another caveat is the misperception that antiplatelets (AP) are somewhat effective and definitively safer than AC rendering them an attractive “soft option” in terms of a safety-driven prescribing attitude [4].

Evaluation of real-world practice is necessary to identify pertinent treatment gaps and to improve provided healthcare services. In this context, we performed a real-world study to assess the type, dosage and predictors of antithrombotic treatment in a representative, nationwide sample of older patients over 80 years with non-valvular atrial fibrillation (NVAF). Reasons for AP use and age subgroup (≥90 and < 90 years) differences were also investigated.

## Methods

### Study population

The PAVE-AF (Pattern of Antithrombotic treatment in the Very Elderly with Atrial Fibrillation) study was a prospective, multicenter, observational study that recruited consecutive patients ≥80 years with NVAF from 30 participating centers in Greece (15 hospitals and 15 private practice centers). The study protocol conformed to the ethical guidelines of the 1975 Declaration of Helsinki and was approved by the respective institutional review boards. Outpatient hospital clinics (university or state, located in either major urban centers or small towns) and private practice cardiologists were included based on their geographical distribution and in an attempt to obtain a representative sample of treating physicians. In order to maintain a nationwide, representative sample, the number of consecutively enrolled patients in each geographical region in Greece was calculated as a percentage share of the total sample size. The respective percentages were defined based on the distribution of the resident population aged ≥ 80 years by geographical region, according to the latest revision of the 2011 Population - Housing Census in Greece as completed by Hellenic Statistical Authority (supplementary Fig. 3, supplementary Table S3) [5].

Inclusion criteria were: (a) male or female patients with confirmed NVAF (excluding moderate to severe mitral stenosis or mechanical prosthetic valves) and (b) age ≥ 80 years. All patients signed an informed consent before their enrolment. AF had to be documented either on surface ECG or on Holter recording with a minimum duration of 30 s. AF definition and classification were standardized based on the latest ESC guidelines [6].

Data were recorded in an electronic case report form (eCRF) and included socio-demographic information, comorbidities and type of treatment. Uncontrolled hypertension was documented in the presence of systolic blood pressure > 160 mmHg, while labile INR was defined as unstable INRs or time in therapeutic range < 60%. Furthermore, the most recent blood test of creatinine levels (within 3 months) was recorded and creatinine clearance (CrCl) was calculated based on the Cockroft-Gault equation. Patients’ actual body weight was included in the respective formula. If patients received either AP only or no treatment for stroke prevention, then physicians were asked to select the reason(s) that led to this decision.

Patients treated with NOACs were classified into three dosing categories (recommended, underdosing and overdosing). Based on the approved European labels, the recommended dosing regimens were the following: *Apixaban:* 5 mg twice daily (bid); dose reduction to 2.5 mg bid if CrCl 15–29 ml/min, or if at least two out of three criteria fulfilled: age ≥ 80 years, body weight ≤ 60 kg, creatinine ≥1.5 mg/dL; contraindicated if CrCl <15 ml/min. *Rivaroxaban*: 20 mg once daily (od); dose reduction to 15 mg od if CrCl 15–49 ml/min; contraindicated if CrCl <15 ml/min. *Dabigatran* 110 mg or 150 mg bid if CrCl >30 ml/min; 110 mg bid in patients ≥80 years or at high risk of bleeding based on the summary of product characteristics; contraindicated if CrCl <30 ml/min.

Since included patients were ≥ 80 years, all apixaban-treated patients met one criterion for dose reduction, while the recommended dose for all dabigatran-treated patients was 110 mg bid. Patients with a contraindication to the administered NOAC due to reduced creatinine clearance levels were classified in the “overdosing” category. All data were acquired during a single visit by the investigator of each center. No follow-up visits were performed.

### Statistics

We calculated that a sample size of 1020 patients would provide statistical power in the range of 88% to 98% with 5% type I error to accurately predict OAC prescription. Descriptive methods were used to characterize the distribution of continuous variables. For non-symmetrically distributed data, the median and interquartile range were calculated. Comparisons were performed using two sample t-test, ANOVA or Mann-Whitney test, as appropriate. For multiple comparisons a Kruskal-Wallis test was applied. Multivariate logistic regression analysis was conducted. In the case of the model selection procedures, the backward selection procedure was performed; an alpha value (level of significance) greater than 10% was used as exclusion criterion. Categorical variables were described by frequencies and related percentages per class level. Comparisons were performed using chi-square test.

## Results

### Antiplatelet therapy or no treatment for stroke prevention

The study population included 1018 patients [mean (SD) 85.4 (4.0) years, 54.4% females], with NVAF (paroxysmal 32.4%, persistent 8% and permanent 59.6%). In total, 899 (88.4%) patients received AC, 82(8%) were treated only with AP, while 37 (3.6%) patients received no treatment. Regarding AP treatment, 37 patients received aspirin, 32 clopidogrel, 2 triflusal, while 11 patients were treated with a combination of aspirin and clopidogrel. Patient characteristics in each treatment group are presented in Table 1.

**Table 1:** Patient characteristics per treatment group

Characteristic	Treatment group			p-value
	Anticoagulant (n = 899)	Antiplatelet only (n = 82)	No treatment (n = 37)	
Age (SD)	85.1 (3.8)	87.5 (5.0)	86.1 (4.2)	< 0.001
Female gender (%)	53.4	62.2	62.2	0.194
Weight (SD)	74.8 (12.6)	73.3 (11.5)	68.2 (13.8)	0.004
Hypertension (%)	78.8	79.2	48.6	< 0.001
Diabetes (%)	28.6	36.6	24.4	0.259
Heart failure^a^ (%)	35.8	33.0	24.4	0.333
Prior stroke/systemic embolism (%)	17.2	19.6	13.6	0.721
Vascular disease^b^ (%)	25	29.2	16.2	0.314
Active smoker^c^ (%)	7.6	6.0	8.2	0.868
Increased alcohol consumption^d^ (%)	4.4	6.0	0.0	0.317
Uncontrolled hypertension^e^ (%)	8.4	17.0	8.2	0.03
Pulmonary disease (%)	21.0	18.2	24.4	0.742
Prior bleeding or high bleeding risk (%)	17.2	26.8	48.6	< 0.001
Coronary heart disease (%)	24.4	28.0	10.8	0.117
Angioplasty/stenting within prior year (%)	2.6	3.6	0.0	0.504
CHADSVASc score (SD)	4.6 (1.4)	4.8 (1.2)	4.0 (1.4)	0.009
HAS-BLED score (SD)	2.4 (0.9)	3.0 (1.0)	2.4 (0.9)	< 0.001

*SD* standard deviation

^a^Heart failure: signs/symptoms of heart failure and/or left ventricular ejection fraction < 40%

^b^Vascular disease: history of myocardial infarction or peripheral arterial disease or presence of atherosclerotic plaque in the aorta

^c^Active smoker: more than two cigarettes daily for at least 2 years

^d^Increased alcohol consumption: more than 8 drinks per week

^e^Uncontrolled hypertension: resting systolic blood pressure > 160/90 mmHg

The type of administered antithrombotic treatment differed significantly among AF type groups with a higher rate of AP treatment among paroxysmal AF patients (supplementary Fig. 4). Furthermore, the type of antithrombotic treatment differed significantly among age subgroups with a lower proportion of nonagenarians receiving anticoagulants (Table 2). Nonagenarians were less likely to receive an AC (OR 0.472) and significantly more likely to receive AP treatment compared to patients < 90 years (OR 2.333, p = 0.001). Similarly, more patients aged ≥9 0 years received no treatment versus patients < 90 years (data not shown).

**Table 2:** Type of antithrombotic treatment administered in different age subgroups

Antithrombotic treatment	Age subgroup			p value
	80–84 years (n = 496)	85–89 years (n = 357)	≥ 90 years (n = 165)	
Anticoagulants	452 (91.2%)	314 (88%)	133 (80.6%)	0.005
Antiplatelet monotherapy	28 (5.6%)	30 (8.4%)	24 (14.6%)	
No treatment	16 (3.2%)	13 (3.6%)	8 (4.8%)	

**Fig. 1:**
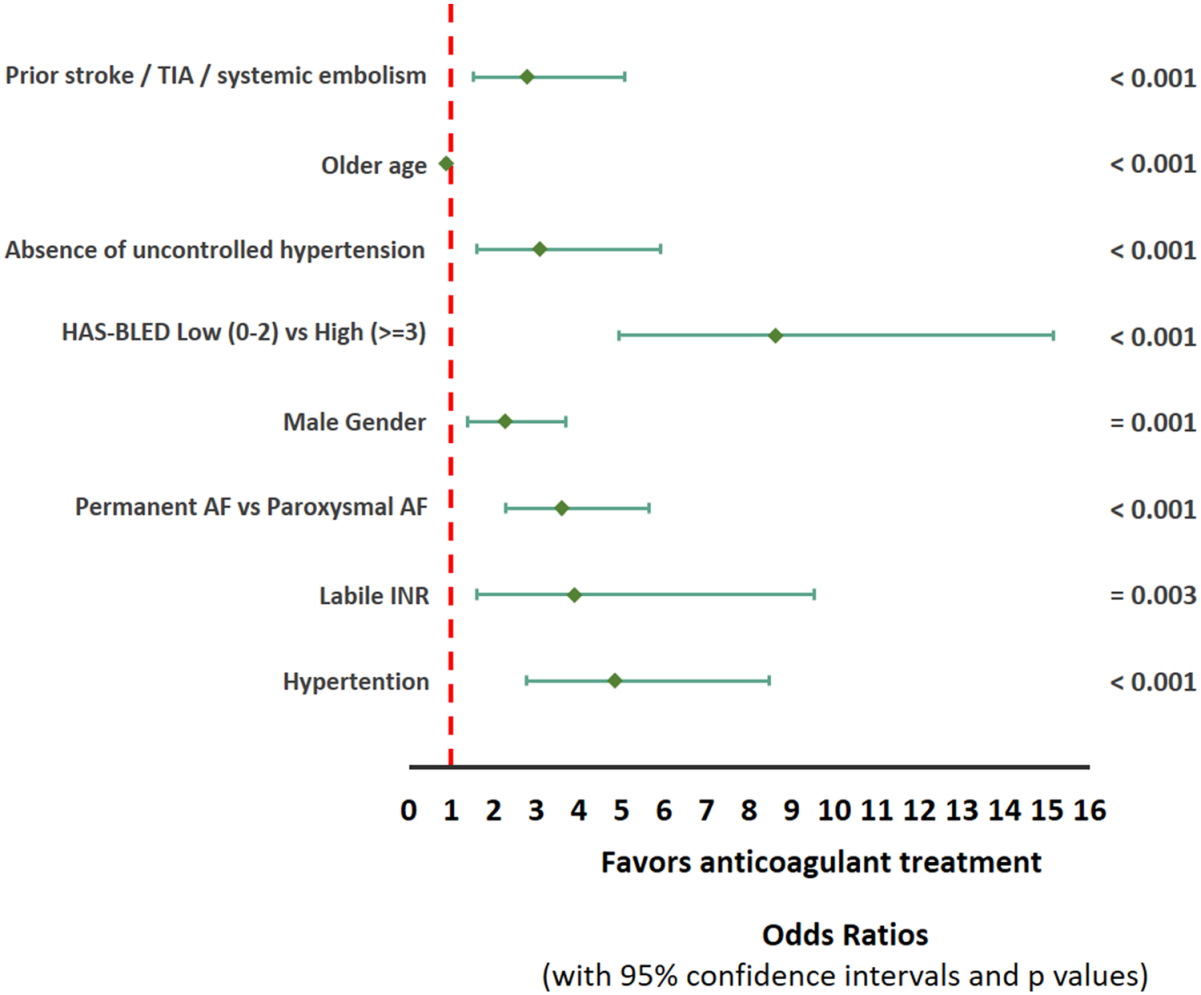
Multivariate predictors of treatment with anticoagulants versus antiplatelets or no treatment

In the multivariate analysis, the following factors were significantly associated with administration of AC treatment as compared to AP or no treatment: low (0–2) HAS-BLED score [OR 8.62, p < 0.001], hypertension [OR 4.85, p < 0.001], labile INR [OR 3.89, p = 0.003] permanent AF [OR 3.60, p < 0.001], absence of uncontrolled hypertension [OR 3.07, p < 0.001], prior stroke/systemic embolism [OR 2.78, p < 0.001], age (continuous variable – per year of age) [OR 0.87, p < 0.001] and male gender [OR 2.26, p = 0.001] (Fig. 1).

### Reasons for antiplatelet monotherapy or no treatment

Physicians reported that the following reason(s) led to their decision to administer either AP only or no treatment for stroke prevention: anticipated high bleeding risk in 57 (31.2%) patients, patient denial in 56 (30.6%), inability of regular INR monitoring in 41 (22.4%), adverse event on prior anticoagulant treatment in 23(12.6%), inability to maintain INR within therapeutic range in 22 (12%), high treatment cost in 10 (5.4%) and other reasons in 20 (11%) patients.

### Type and dosage of anticoagulant treatment

**Fig. 2:**
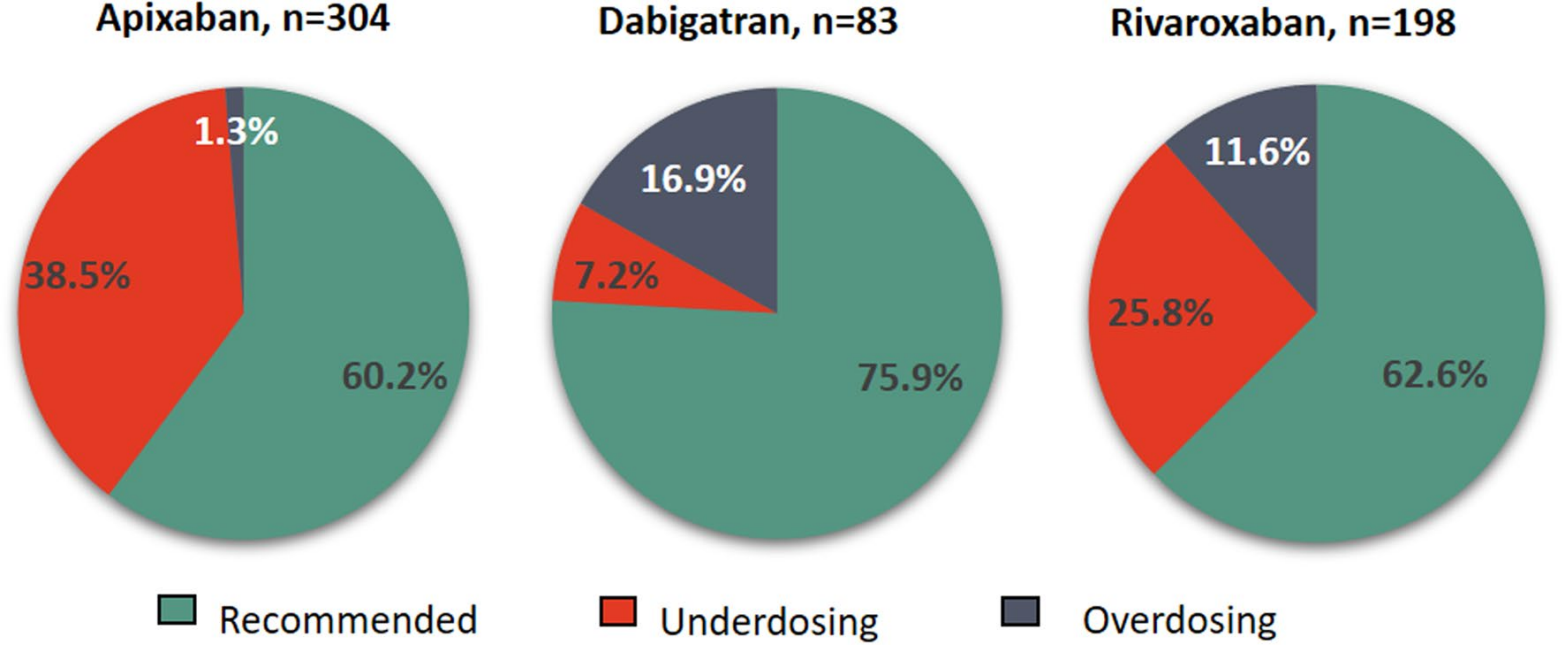
Percentage of NOAC recipients receiving recommended dosing, overdosing or underdosing

Among 899 patients treated with anticoagulants, 21 received parenteral and 878 oral antithrombotic treatment. In the latter subgroup, 293 patients were treated with vitamin K antagonists (33.4%), while 585 (66.6%) received NOACs [304 (34.6%) apixaban, 83 (9.5%) dabigatran and 198 (22.6%) rivaroxaban]. A significantly lower percentage of patients ≥ 90 years were treated with NOACs as compared to patients aged 80–89 years (48.4% vs 59.2%, respectively, p < 0.05). The reduced NOAC doses were administered in 71.8% of apixaban-treated patients (2.5 mg bid), in 82% of dabigatran-treated patients (110 mg bid) and in 63.2% of rivaroxaban-treated patients (15 mg od). In total, 63.2% of patients received NOAC dosing consistent with European label recommendations, 29.7% received a lower dose, while 7.1% were overdosed (supplementary Table S4). The percentage of patients receiving recommended, over- or under- dosing differed significantly among the three NOAC groups (p < 0.001), (Fig. 2).

## Discussion

The main finding of this prospective, nationwide, representative, real-world study is that 11.6% of patients > 80 years with NVAF receive antiplatelet monotherapy or no treatment for stroke prevention. Among patients treated with oral anticoagulants, two thirds are under NOAC treatment. However, only 63.2% of NOAC recipients receive appropriate dosing based on European label recommendations.

### Antiplatelet treatment for stroke prevention

The latest ESC guidelines do not recommend antiplatelet monotherapy for stroke prevention in AF patients [6]. However, there has always been a considerable time lag before issued recommendations are incorporated into everyday practice. Several real-world surveys have reported the persistence of antiplatelet use for stroke prevention in AF patients [7–10]. However, most registries do not provide detailed data regarding prescription patterns in older patients. Furthermore, reported findings pertain to a transient period following approval and initial market penetration of different NOACs. This dynamic state may have prevented the capture of a steady and representative prescription behavior of physicians for stroke prevention. Our study enrolled patients several years post launch of NOACs in the Greek market, where the existing pricing and reimbursement policy allows patient access to this pharmacological treatment, thus reducing potential confounding effect of drug availability and affordability. In our study we found that 8% of real-world AF patients over 80 years are treated only with antiplatelets for stroke prevention, while only 3.6% receive no treatment at all. The most frequently reported reasons why an anticoagulant was not administered included anticipated high bleeding risk, patient denial and inability of regular INR monitoring, while treatment cost influenced therapeutic choices in a minority of cases. Our study also demonstrated several independent predictors associated with administration of antiplatelet monotherapy or no treatment in older patients over 80 years with AF, including increased bleeding risk (high HAS-BLED score), older age, uncontrolled hypertension, female gender and history of paroxysmal AF.

### NOAC type and dosing in older AF patients

NOACs have evidence-based credentials for stroke prevention in patients with NVAF [11]. Their advent has signaled a shift in the prescribing pattern of therapies for stroke prevention. A Danish nationwide registry showed a significant decrease in the usage of vitamin K antagonists from 92 to 24%, with a counterbalancing increase in NOAC use [12]. Real world data evaluating the use of NOACs in older AF patients are scarce. In our study, two thirds of patients treated with oral anticoagulants received NOACs, thus supporting increased utilization of NOACs over vitamin K antagonists.

Apart from the standard doses, reduced NOAC dosing schemes have been approved for predefined patient subgroups with certain baseline characteristics [11]. The administration of reduced NOAC dosing in patients not meeting the recommended criteria worsens patient outcome and significantly increases the risk of adverse events [13]. NOAC overdosing was reported to increase all-cause mortality, while underdosing increased cardiovascular hospitalizations as compared to recommended doses [13]. The reported incidence of inappropriate NOAC dosing varies. Steinberg et al. have reported that only 13% of AF patients treated with NOACs received off-label dosing [13]. This percentage is considerably lower than the respective in our study. We found that 29.7% of patients received a lower dose, while 7.1% were overdosed. Several factors may explain this discrepancy. The authors have commented that the reported results may underestimate the magnitude of incorrect dose selection in broader AF populations, due to the potential confounding effect of participation in an AF registry [13]. The ORBIT-AF II registry evaluated the US community practice and ascertainment of appropriate NOAC dosing was based on the FDA-approved package inserts that differ from the respective European labels. Thus, the use of different classification criteria limits the extrapolation of relevant findings in the European care setting. Furthermore, the ORBIT-AF II analysis included a general AF patient population, with a mean age of 70 years, while our study specifically focused in the older patient subpopulation over 80 years. The popularity of a “softer” anticoagulant prescribing practice among AF patients over 80 years due to concerns on frailty, bleeding and falling, may partly explain the increased rate of underdosing [14]. In line with our results, Barra et al., in a retrospective chart review of 224 patients with a mean age of 77.7 years who received a reduced NOAC dose, found that only 43.3% of patients satisfied the dose adjustment criteria recommended by the manufacturer labeling [15].

Our findings reflect physicians’ willingness to avoid bleeding complications in the context of a safety-driven decision-making in anticoagulant type and dosage. Interestingly, there is a shift in the adopted “soft” thromboprotective options from antiplatelet monotherapy to inappropriately low doses of NOACs. However, inappropriate NOAC dosing in the older AF patients still deprives them of optimal thromboprotection. Intensive educational campaigns are needed to further familiarize prescribing physicians with the indicated NOAC dosing schemes and to educate them on the unfavorable prognostic impact of inappropriate NOAC dosing.

### Strengths and limitations

Several methodological strengths of our study should be commented. PAVE-AF is a prospective non-interventional study which is considered to provide evidence of “higher quality” than retrospective claims analyses. The latter is a common source of real-world evidence, but prone to bias due to void selection of diagnosis coding, missing data and lenient case definition. In our study, AF was confirmed either by surface ECG or on Holter monitoring with a duration of at least 30 s. Furthermore, we used minimal exclusion criteria to increase the external validity of our results. The study population had a representative geographical distribution to reduce the likelihood of systematic enrolment bias and to increase the generalizability of our findings. The number of patients enrolled in each geographical region was proportional to the reference population (≥80 years), based on the latest national population census. The participating centers have been selected on the basis of including different care setting for AF patients including both physician offices, as well as different types of hospitals. In addition, consecutive sampling was employed with a predefined target size to enhance the representativeness of the reference population and all eCRFs were validated through source data verification processes. However, strict implementation of consecutive patient enrolment cannot be confirmed, since use of screening logs was not mandated by the study protocol; thus, selection bias cannot be excluded. Another limitation of the study is the lack of follow-up data that do not allow the evaluation of patient outcome.

#### Clinical implication

The results of our real-world study contribute to the identification of antithrombotic treatment pattern in older NVAF patients, a population with limited data in the literature. Based on our findings, older patients with NVAF are mostly treated with anticoagulants and preferably with a NOAC. However, nonagenarians are twice likely to receive an AP or no treatment mainly due to physician’s fear of bleeding. Most importantly, more than one third of our population receive inappropriate dosage. Although our results are encouraging and partly imply adherence to current treatment guidelines, they raise a red flag with respect to inappropriate dosage, pointing out the need for continuous education on appropriate drug therapy irrespective of patient’s age.

## Conclusion

In the real world, a minority of older patients > 80 years with non-valvular AF receives antiplatelet monotherapy or no treatment for stroke prevention. Among patients treated with oral anticoagulants, two thirds are on NOAC treatment, but inappropriate dosing remains a significant barrier preventing optimal patient thromboprophylaxis.

## Electronic supplementary material

Below is the link to the electronic supplementary material.

Electronic supplementary material 1 (DOCX 615 kb) Geographical distribution and treatment of study population
