# Supplementary material for: Right drug, wrong dosage: insights from the PAVE-AF antithrombotic study in older patients with atrial fibrillation
Source: J Thromb Thrombolysis. 2020 Jun 9;51(1):81–8. doi: 10.1007/s11239-020-02167-8 (PMC7829237; doi:10.1007/s11239-020-02167-8)

**Supplementary Table S3**

Distribution of the resident population aged 80 years and older by region, according to the latest revision of the 2011 Population - Housing Census in Greece as completed by Hellenic Statistical Authority and respective number of patients enrolled in the study.

| **Region** | **Population aged ≥ 80 years (2011 Census)** | **Percentage share per region** | **Number of patients enrolled (population size 1018 patients)** |
| --- | --- | --- | --- |
| 1. East Macedonia and Thrace | 38731 | 7% | 71 |
| 1. Central Macedonia | 74099 | 13,4% | 137 |
| 1. West Macedonia | 24395 | 4,4% | 45 |
| 1. Epirus | 31076 | 5,6% | 57 |
| 1. Thessaly | 48177 | 8,7% | 89 |
| 1. Central Greece | 46816 | 8,5% | 86 |
| 1. Ionian islands | 20422 | 3,7% | 38 |
| 1. West Greece | 51536 | 9,3% | 95 |
| 1. Peloponnese | 63632 | 11,5% | 117 |
| 1. Attica | 71241 | 12,9% | 131 |
| 1. North Aegean islands | 22916 | 4,1% | 42 |
| 1. South Aegean islands | 21001 | 3,8% | 39 |
| 1. Crete | 38891 | 7% | 71 |

**Supplementary Table S4.**

Distribution of dosing regimens among patients receiving NOACs^a^ and classification in dosing groups: (a) recommended dosing, (b) underdosed, (c) overdosed based on European label recommendations.

| **Apixaban (n=304)** | | | | | | | | |
| --- | --- | --- | --- | --- | --- | --- | --- | --- |
| **Recommended dosing** | | | **Underdosed** | | | **Overdosed** | | |
| **Dosing schema** | **N** | **%** | **Dosing schema** | **N** | **%** | **Dosing schema** | **N** | **%** |
| 2.5mg bid, CrCl:15–29 ml/min | 42 | 13.8 | 2.5 mg bid, in the absence of **both** Cr ≥ 1.5 mg/dL and weight ≤ 60 kg | 116 | 38.2 | 5 mg bid, Cr ≥ 1.5 mg/dL | 2 | 0.7 |
| 2.5 mg bid, Cr ≥ 1.5 mg/dL | 19 | 6.3 |  |  |  | 5 mg bid, weight ≤ 60 kg | 2 | 0.7 |
| 2.5 mg bid, weight ≤ 60 kg | 41 | 13.5 | 2.5 mg o.d. | 1 | 0.3 |  |  |  |
| 5 mg bid, in all other patients | 81 | 26.6 |  |  |  |  |  |  |
| **Dabigatran (n=83)** | | | | | | | | |
| **Recommended dosing** | | | **Underdosed** | | | **Overdosed** | | |
| **Dosing schema** | **N** | **%** | **Dosing schema** | **N** | **%** | **Dosing schema** | **N** | **%** |
| 110 mg bid | 63 | 75.9 | 75mg bid | 4 | 4.8 | 150mg bid | 9 | 10.8 |
|  |  |  | 110mg od | 1 | 1.2 | Any dose if CrCl<30 ml/min | 5 | 6.0 |
|  |  |  | 150mg od | 1 | 1.2 |  |  |  |
| **Rivaroxaban (n=198)** | | | | | | | | |
| **Recommended dosing** | | | **Underdosed** | | | **Overdosed** | | |
| **Dosing schema** | **N** | **%** | **Dosing schema** | **N** | **%** | **Dosing schema** | **N** | **%** |
| 15 mg od, CrCl:15-49 ml/min | 74 | 37.4 | 15 mg od, CrCl≥50 ml/min | 50 | 25.3 | 20 mg od, CrCl:15-49 ml/min | 22 | 11.1 |
| 20 mg od, CrCl ≥ 50 ml/min | 50 | 25.3 | 10mg od | 1 | 0.5 | Any dose if CrCl < 15 ml/min | 1 | 0.5 |

^a^non-vitamin K oral anticoagulants

**Supplementary Figure 3**

Μap of Greece with all regions reporting the percentage share and number

of patients enrolled in the study.


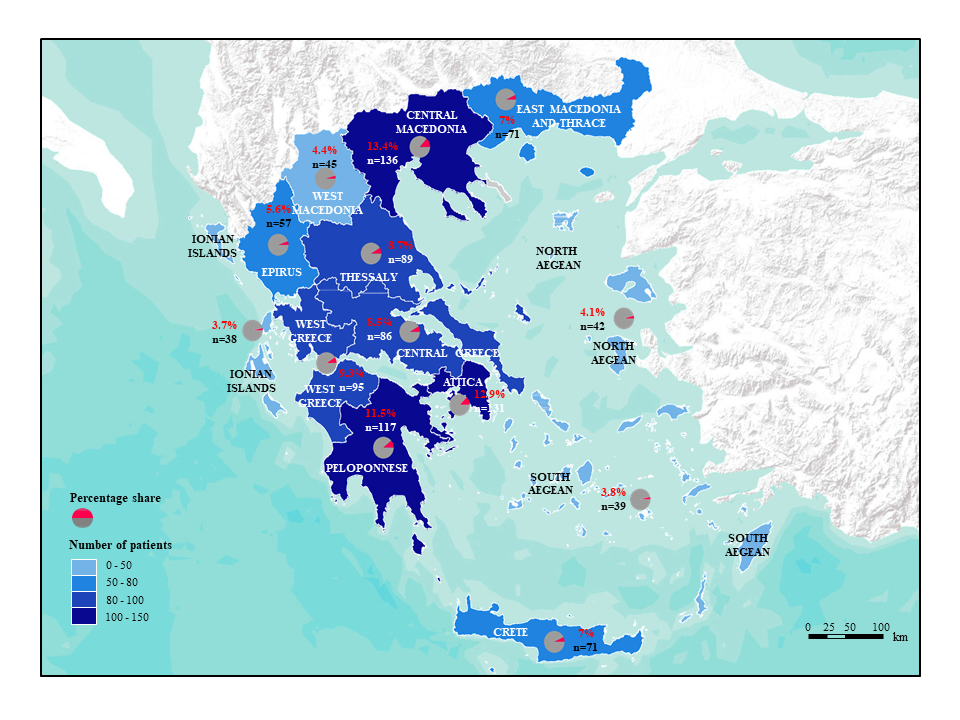


**Supplementary Figure 4**

Type of anticoagulant treatment administered in different atrial fibrillation type groups.


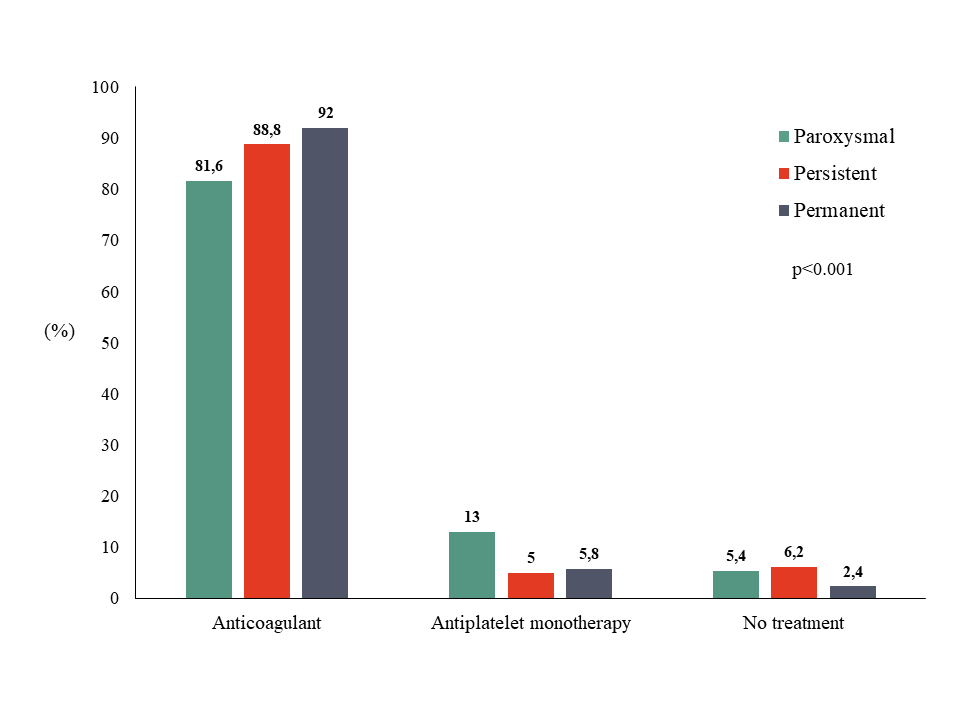

Supplement: Supplementary file 1 — Electronic supplementary material 1 (DOCX 615 kb) Geographical distribution and treatment of study population [file 11239_2020_2167_MOESM1_ESM.docx]
